# Supplementary material for: Checkpoint inhibition through small molecule-induced internalization of programmed death-ligand 1
Source: Nat Commun. 2021 Feb 22;12:1222. doi: 10.1038/s41467-021-21410-1 (PMC7900207; doi:10.1038/s41467-021-21410-1)
Supplement: Supplementary file 1 — Supplementary Information [file 41467_2021_21410_MOESM1_ESM.pdf]

Supplementary Figure 1

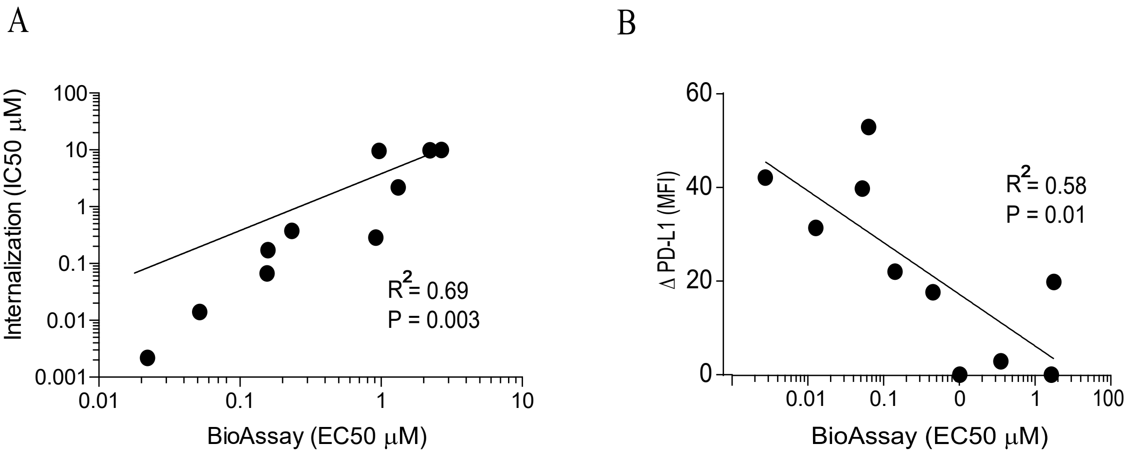

BioAssay, HTRF, and Internalization assay correlations
(a) Correlation between IC_{50} values by internalization assay and EC_{50} values by bioassay of n=10 compounds. Correlation was done by Pearson's test. (b) Correlation between cell surface PD-L1 levels (MFI) and EC50 values by bioassay of n=10 compounds. Correlation was done by Pearson's test. Source data are provided as a source data file.

Supplementary Figure 2

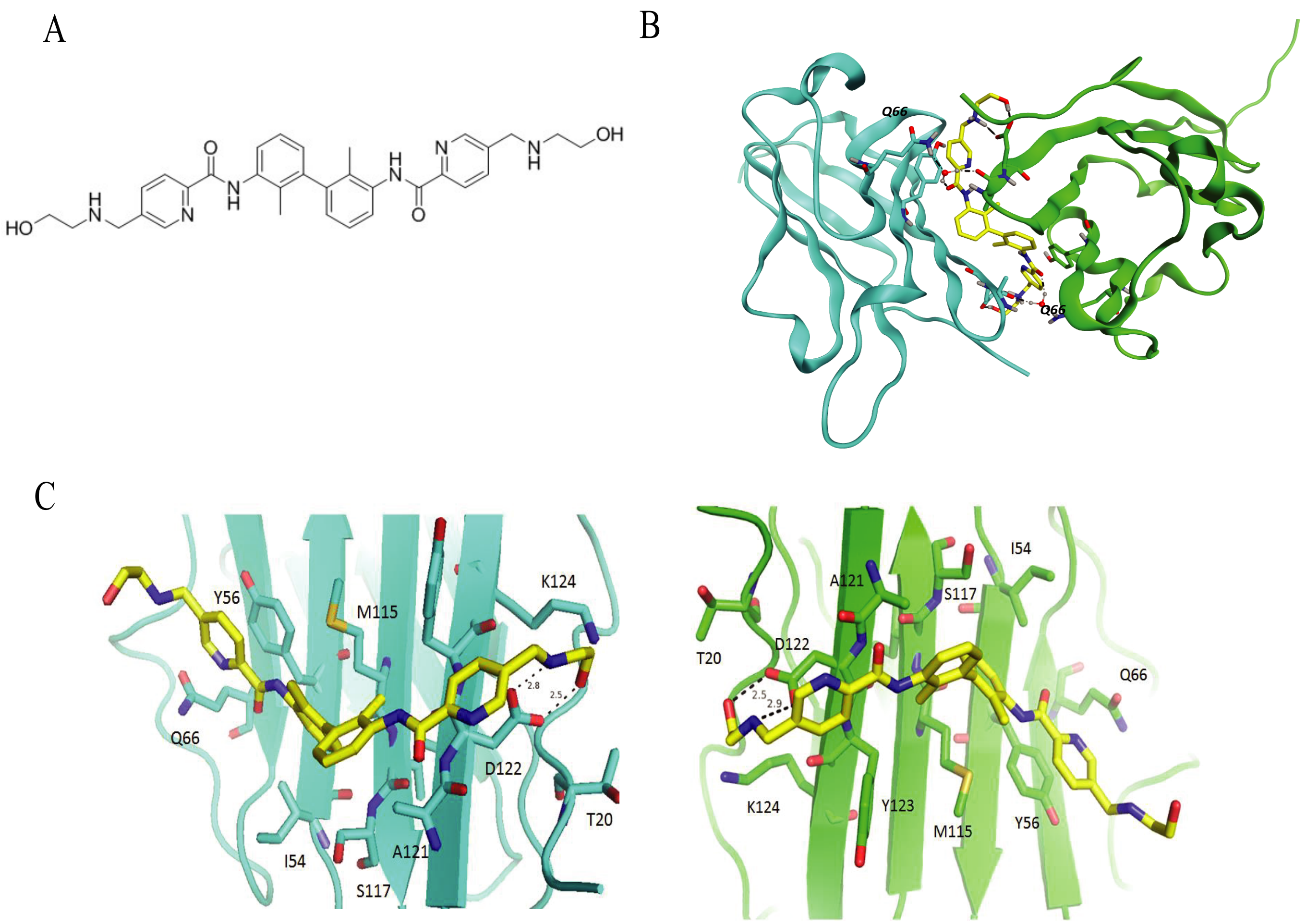

Co-crystal X-ray structure of compound A and PD-L1 protein
(a) Structure of compound A (b) Fit of compound A at dimeric PD-L1 interface. The complex structure was resolved at 2.49 Angstroms. PD-L1 in ribbon are colored cyan and green for chains A and B, respectively. Compound A is shown as a stick with carbon in magenta, oxygen in red and nitrogen in blue. (c) Interaction of compound A with Chain A (left panel) and with Chain B (right panel): Stick diagram with Chain A (carbon atoms colored green, oxygen red, nitrogen blue and sulfur yellow), and with Chain B (carbon atoms colored cyan, oxygen red, nitrogen blue and sulfure yellow). Compound A is shown as a stick figure with yellow carbon atoms, oxygen red and nitrogen blue. Source data are provided as a source data file.

Supplementary Figure 3

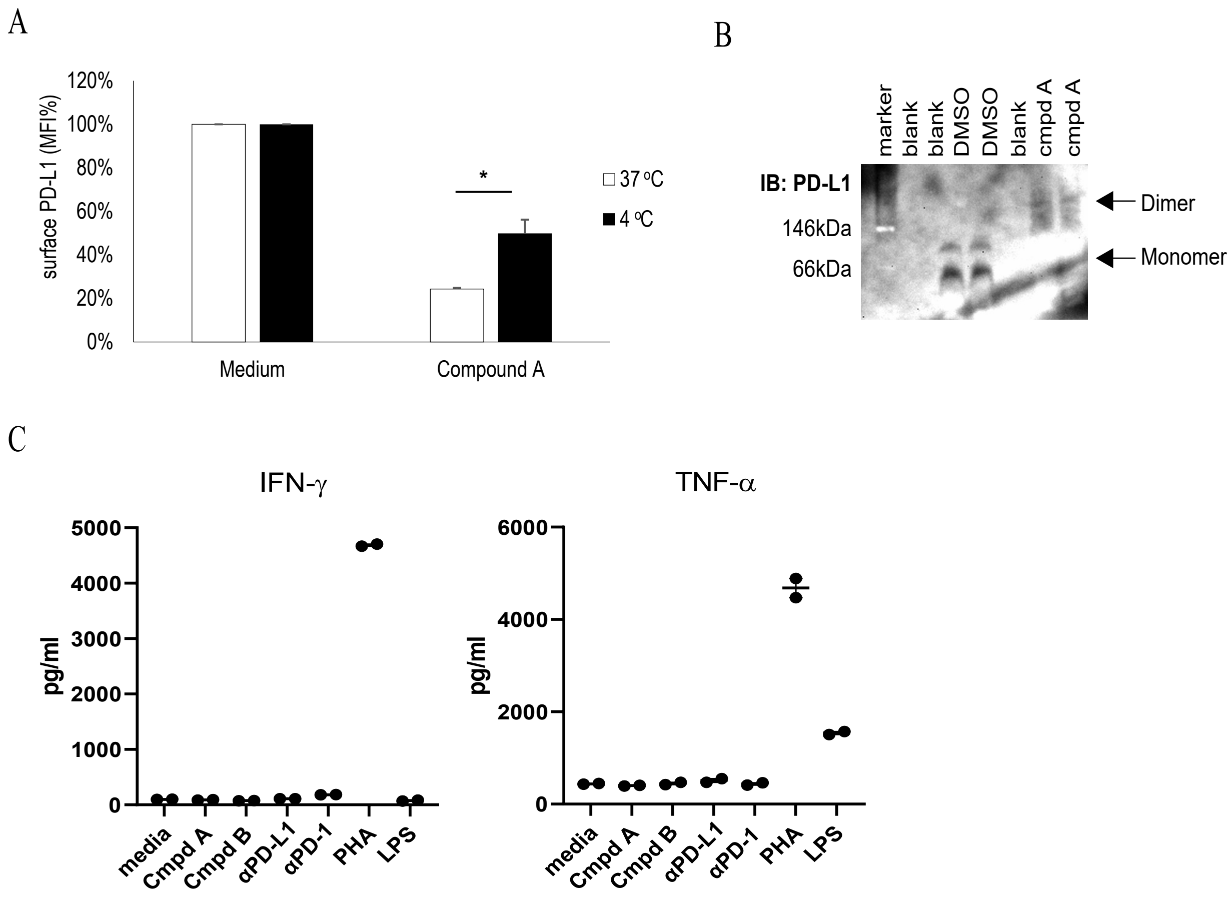

Effect of temperature on internalization

(a) Effect of compound A on surface PD-L1 internalization in 4°C and 37°C (b) Native gel electrophoresis of PD-L1 protein with and without compound A treatment to CHO-PD-L1 cells (c) Cytokine quantification in human PBMC supernatant following stimulation with compound A, compound B, αPD-1, αPD-L1, and positive controls PHA and LPS. IFN-γ and TNF-α cytokine expression from PBMCs are represented. Source data are provided as a source data file.

Supplementary Figure 4

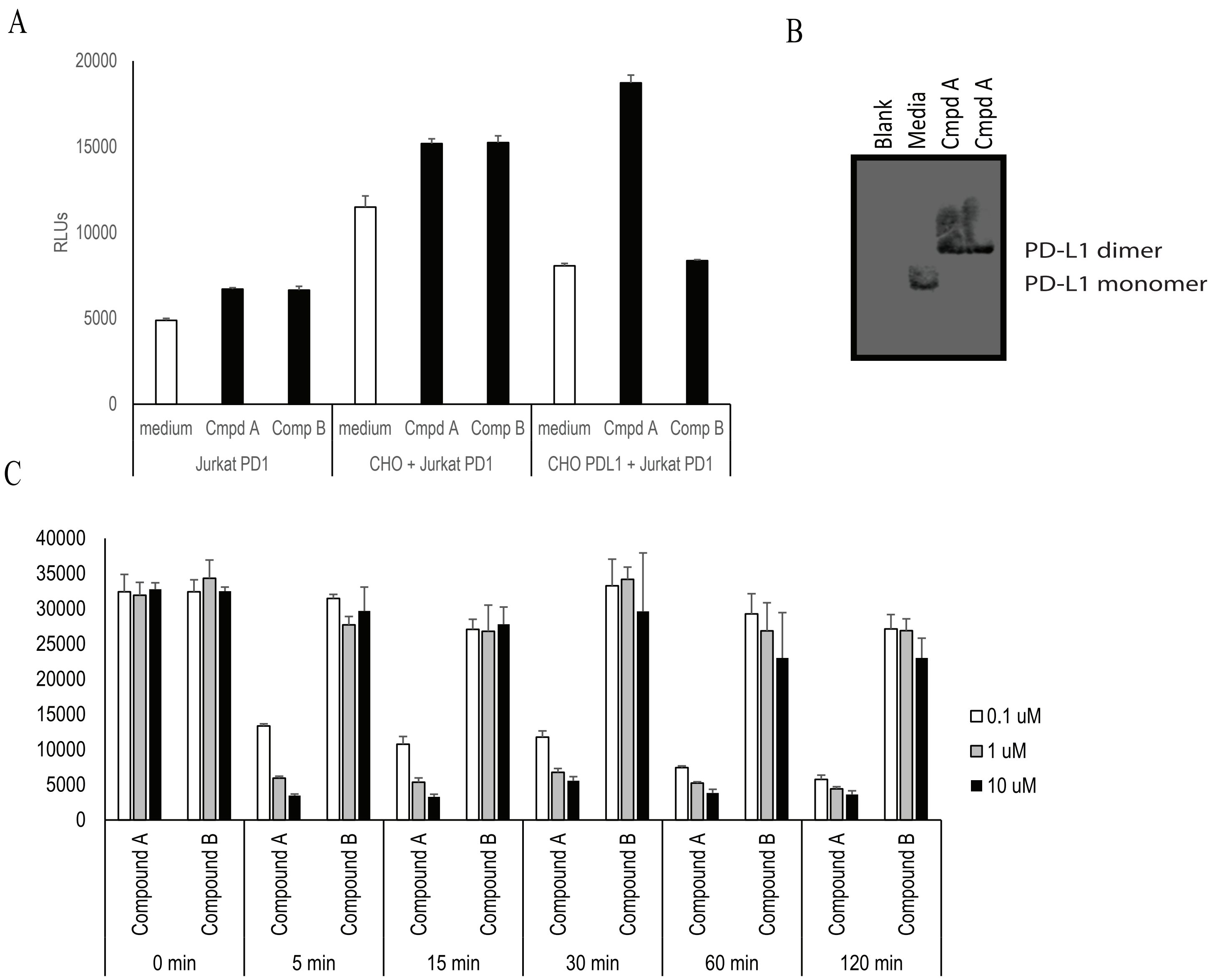

Dose response of small molecule-induced PD-L1 internalization
(a) Cell surface PD-L1 expression (RLUs) in Jurkat PD-1, CHO knockout PD-L1 + Jurkat PD-1 co-culture, and CHO PD-L1 + Jurkat PD-1 co-cultures following treatment with compound A or compound B (b) Immunoblot of recombinant PD-L1 protein with and without compound A treatment. Monomeric and dimeric PD-L1 protein are notated (c) Kinetics of compound A-induced PD-L1 cell surface internalization. Source data are provided as a source data file.

Supplementary Figure 5

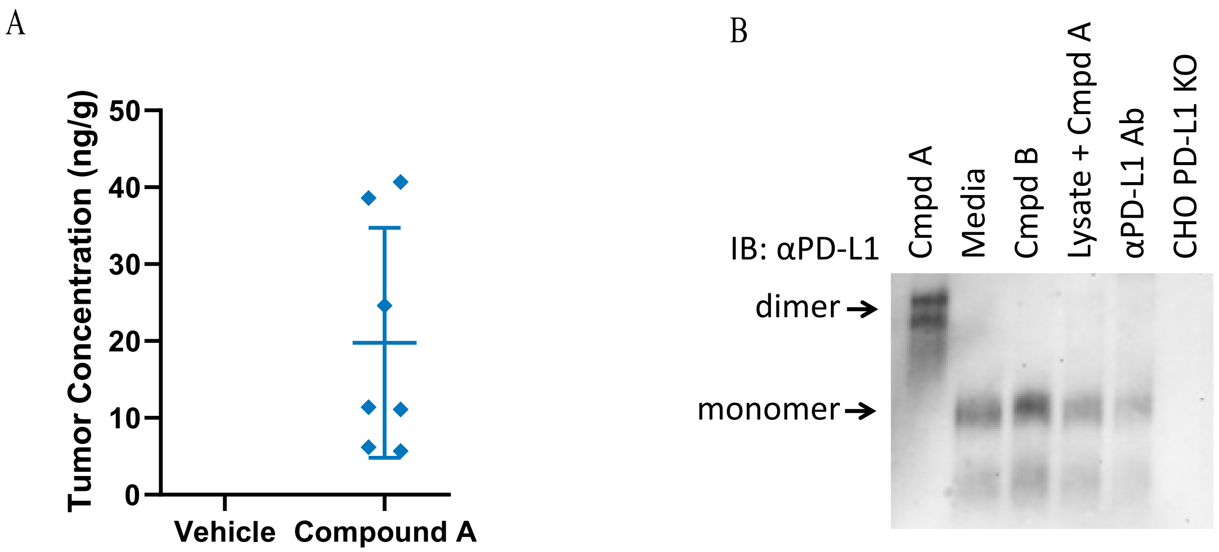

Compound A tumor concentrations

(a) Tumor compound A concentrations (ng/g) on day 28 (study termination) (b) Native PD-L1 immunoblot in cells devoid of PD-L1 (CHO PD-L1 KO) with and without compound A treatment. Dimer and monomer are indicated with an arrow. Source data are provided as a source data file.

Supplementary Table 1 Data Collection and Refinement Statistics

	PD-L1/Compound A Complex
**Data Collection**	
Space Group	$C222_1$
Cell Dimensions<br>a, b, c (Å)<br>α, β, γ (°)	 99.003, 172.173, 80.585<br>90, 90, 90
Resolution (Å)	80.59 – 2.49 (2.62 – 2.49)*
R_{merge}	9.7 (116)*
$I/\sigma I$	14.6 (2.0)*
Completeness (%)	99.1 (99.1)*
Redundancy	7.3 (7.7)*
**Refinement**	
Resolution (Å)	2.49
No. reflections	23,114
R_{work}/R_{free}	0.177/0.228
No. atoms<br>Protein<br>Ligand<br>Waters	 2,912<br>63<br>139
Average *B*-factors	A, B, C
main chain atoms<br>side chain atoms<br>Ligand<br>Water	53.0, 54.2, 55.6<br>61.4, 63.2, 66.3<br>47.2(A:B), 45.6(C:C')<br>56.3
R.M.S.D.§<br>Bond Lengths (Å)<br>Bond Angles (°)	 0.012<br>1.66

*Values in parentheses represent the highest resolution shell.
§Root-mean square deviation from target geometry.
