# Supplementary material for: Checkpoint inhibition through small molecule-induced internalization of programmed death-ligand 1
Source: Nat Commun. 2021 Feb 22;12:1222. doi: 10.1038/s41467-021-21410-1 (PMC7900207; doi:10.1038/s41467-021-21410-1)
Supplement: Supplementary file 3 — Reporting Summary [file 41467_2021_21410_MOESM3_ESM.pdf]

nature research

Corresponding author(s): Chris B. Moore, PhD

Last updated by author(s): 12/07/2020

Reporting Summary

Nature Research wishes to improve the reproducibility of the work that we publish. This form provides structure for consistency and transparency in reporting. For further information on Nature Research policies, see our Editorial Policies and the Editorial Policy Checklist.

Statistics

For all statistical analyses, confirm that the following items are present in the figure legend, table legend, main text, or Methods section.

n/a	Confirmed	
☐	☒	The exact sample size (n) for each experimental group/condition, given as a discrete number and unit of measurement
☒	☐	A statement on whether measurements were taken from distinct samples or whether the same sample was measured repeatedly
☐	☒	The statistical test(s) used AND whether they are one- or two-sided<br>*Only common tests should be described solely by name; describe more complex techniques in the Methods section.*
☒	☐	A description of all covariates tested
☒	☐	A description of any assumptions or corrections, such as tests of normality and adjustment for multiple comparisons
☐	☒	A full description of the statistical parameters including central tendency (e.g. means) or other basic estimates (e.g. regression coefficient) AND variation (e.g. standard deviation) or associated estimates of uncertainty (e.g. confidence intervals)
☐	☒	For null hypothesis testing, the test statistic (e.g. F, t, r) with confidence intervals, effect sizes, degrees of freedom and P value noted<br>*Give P values as exact values whenever suitable.*
☒	☐	For Bayesian analysis, information on the choice of priors and Markov chain Monte Carlo settings
☒	☐	For hierarchical and complex designs, identification of the appropriate level for tests and full reporting of outcomes
☒	☐	Estimates of effect sizes (e.g. Cohen's d, Pearson's r), indicating how they were calculated

Our web collection on statistics for biologists contains articles on many of the points above.

Software and code

Policy information about availability of computer code

Data collection	No software was used
Data analysis	No software was used

For manuscripts utilizing custom algorithms or software that are central to the research but not yet described in published literature, software must be made available to editors and reviewers. We strongly encourage code deposition in a community repository (e.g. GitHub). See the Nature Research guidelines for submitting code & software for further information.

Data

Policy information about availability of data

All manuscripts must include a data availability statement. This statement should provide the following information, where applicable:

- Accession codes, unique identifiers, or web links for publicly available datasets
- A list of figures that have associated raw data
- A description of any restrictions on data availability

Crystal structure data that support the findings of this study have been deposited in RCSB protein database (6VQN.pdb). The authors declare that all other data supporting the findings of this study are available within the paper and its supplementary information files.

Field-specific reporting

Life sciences study design

All studies must disclose on these points even when the disclosure is negative.

Sample size	sample sizes primarily pertain to our animal studies and were determined from historical checkpoint inhibitor anti-tumor studies and based on the minimum sample size to yield a statistical difference from those studies.
Data exclusions	No data was excluded
Replication	All data in this manuscript represents replicated data from three independent experiments. No data is shown that cannot be replicated using the methods described in this manuscript
Randomization	No experiments were performed using clinical subjects therefore randomization does not apply. For our animal studies all mice were randomized into each treatment cohort.
Blinding	Blinding was only necessary in our mouse studies. Each treatment was blinded using random identifiers so that no information was provided to the technicians about the test articles being administered

Reporting for specific materials, systems and methods

We require information from authors about some types of materials, experimental systems and methods used in many studies. Here, indicate whether each material, system or method listed is relevant to your study. If you are not sure if a list item applies to your research, read the appropriate section before selecting a response.

Materials & experimental systems

n/a	Involved in the study	
☐	☒	Antibodies
☐	☒	Eukaryotic cell lines
☒	☐	Palaeontology and archaeology
☐	☒	Animals and other organisms
☒	☐	Human research participants
☒	☐	Clinical data
☒	☐	Dual use research of concern

Methods

n/a	Involved in the study	
☒	☐	ChIP-seq
☐	☒	Flow cytometry
☒	☐	MRI-based neuroimaging

Antibodies

Antibodies used

1. αPD-L1 PE antibody (eBioscience, clone MIH1) 2. Anti-Myc antibody (Clone 9E10)-HRP (Abcam, Cat No. ab62928, 1:10000) 3. Anti-Flag (DDDDK) tag antibody (Clone M2)-HRP (Abcam, Cat No. ab49763, 1:1000), 4. β-Actin Monoclonal antibody (Clone BA3R)-HRP (Invitrogen, Cat No. MA5-15739-HRP, 1:1000)
2. APC/Cy7 anti-CD 20, Biolegend, 302314, clone 2H7
3. Alexa Fluor 700-anti-CD38, Biolegend, 356624, clone HB-7
4. Brilliant Violet 785-anti-CD27, Biolegend, 302832, clone O323
5. Brilliant Violet 650-anti-CD3, Biolgend, 317324, clone OKT3
6. Brilliant Violet 605-anti-IgD, Biolegend, 348232, clone IA6-2

Validation

1. "12-5983 was used in Flow cytometry/Cell sorting to demonstrate that macrophage NOS2 promotes lung squamous cell carcinoma development by circulating inflammation, enhancing macrophage survival, and impairing lipid metabolism." 2. "The laboratory confirmed that they tested 0.1ug E. coli with myc-tag in WB and they obtained a good signal at 1 min exposure. However, they did not test the limits of ab62928 products sensitivity, but they would expect it to be able to detect smaller quantities than they have used." 3. "Western blot abreview for Anti-DDDDK tag (Binds to FLAG® tag sequence) antibody [M2] (HRP)" 4. Western blot analysis was performed on whole cell extracts (30 µg lysate) of A549 (Lane 1), COS-7 (Lane 2), MDCK (Lane 3), C2C12 (Lane 4), MDA-MB-231 (Lane 5), RSC96 (Lane 6) and tissue extracts of Mouse Lung (Lane 7). The blot was probed with beta Actin Monoclonal Antibody (BA3R), HRP (Product # MA5-15739-HRP, 1 µg/mL) and detected by chemiluminescence.
2. Technical data sheet: "Each lot of this antibody is quality control tested by immunofluorescent staining with flow cytometric analysis. Application references: a. Schlossman S, et al. 1995. Leucocyte Typing V. Oxford University Press. New York. b. Knapp W, et al. 1989. Leucocyte Typing IV. Oxford University Press. New York. c. McMichael A, et al. Eds. 1987. Leucocyte Typing III Oxford University Press. New York. d. Polyak MJ, et al. 2002. Blood 99:3256. (IP) e. Mack CL, et al. 2004. Pediatr. Res. 56:79. (IHC)"
3. Technical data sheet: " Each lot of this antibody is quality control tested by immunofluorescent staining with flow cytometric analysis. Product Citations. a. Hoffmann M, et al. 2016. PLoS Pathog. 12: 1005661. PubMed. b. Chen L et al. 2018. Immunity. 49 (3):464-476 . PubMed"
4. Technical data sheet: " Each lot of this antibody is quality control tested by immunofluorescent staining with flow cytometric analysis. a. Knapp W, et al. Eds. 1989. Leucocyte Typing IV. Oxford University Press. New York. b. Correia DV, et al. 2011. Blood 118:992. (FC) PubMed"
5. Technical data sheet: " Each lot of this antibody is quality control tested by immunofluorescent staining with flow cytometric analysis. Product Citations a. Tilton C, et al. 2013. J Virol Methods. 166:376. PubMed b. Boudreau J, et al. 2014. PLoS One. 9:99543.

PubMed c. Riou C, et al. 2014. PLoS One. 9:102178. PubMed"
6. Technical data sheet: " Each lot of this antibody is quality control tested by immunofluorescent staining with flow cytometric analysis. Application References a. Chen K, et al. 2009. Nat. Immunol. 10:889. b. Lee CH, et al. 2005. J. Exp. Med. 203:63. c. Sutter JA, et al. 2008. Clin. Immunol. 126:282.

Eukaryotic cell lines

Policy information about cell lines

Cell line source(s)	Promega's CHO-K1 cells stably expressing human PD-L1 and Jurkat T cells stably expressing human PD-1 and NFAT-induced luciferase. For KO studies, Promega PD-L1 negative cells (aAPC/CHO-K1)
Authentication	1. Flow cytometry confirmed that this cell line does not express PD-L1. 2. PD-L1 expression was confirmed by flow cytometry. 3. This cell line was confirmed by anti-CD3 antibody stimulation. * all 3 cell lines were purchased from Promega with data sheets confirmation.
Mycoplasma contamination	Each cell line was validated mycoplasm free by the vendor
Commonly misidentified lines (See ICLAC register)	*Name any commonly misidentified cell lines used in the study and provide a rationale for their use.*

Animals and other organisms

Policy information about studies involving animals; ARRIVE guidelines recommended for reporting animal research

Laboratory animals	Mouse, C57BL/6-Pdcd1tm1(PDCD1) Cd274tm1(CD274)/Bcgen, female, 7-10 weeks old
Wild animals	Study did not involve wild animals
Field-collected samples	Study did not involve samples collected from the field
Ethics oversight	Mouse study was approved by Biocytogen's Institutional Animal Care and Use Committee and was in compliance with the Guide for the Care and Use of Laboratory Animals (National Research Council, 2011).

Note that full information on the approval of the study protocol must also be provided in the manuscript.

Flow Cytometry

Plots

Confirm that:

- [x] The axis labels state the marker and fluorochrome used (e.g. CD4-FITC).
- [x] The axis scales are clearly visible. Include numbers along axes only for bottom left plot of group (a 'group' is an analysis of identical markers).
- [x] All plots are contour plots with outliers or pseudocolor plots.
- [x] A numerical value for number of cells or percentage (with statistics) is provided.

Methodology

Sample preparation	PBMCs were harvested using Ficoll separation. Cell lines were harvested following treatment with trypsin. PBMCs and cell lines were stained with antibodies and fixed with 2% PFA before acquiring by flow cytometry
Instrument	LSRFortessa was used for acquiring stained samples
Software	FlowJo was used for Flow data analysis.
Cell population abundance	10,000 cells were collected for analysis.
Gating strategy	Forward versus side scatter (FSC vs SSC) gating is commonly used to identify cells of interest based on size and granularity (complexity). The live cells were further gated by Aqua LIVE/DEAD Fixable Stains negative population as live cells.

- [x] Tick this box to confirm that a figure exemplifying the gating strategy is provided in the Supplementary Information.
